# Mapping the Associations Between Body Mass Index and Key Psychosocial Determinants: Resilience, Perceived Stress, and Emotion Regulation in Young Adults—A Cross-Sectional Study

**DOI:** 10.3390/healthcare13233150

**Published:** 2025-12-02

**Authors:** Boris Tilov, Pavel Stanchev, Mariya Dimitrova, Meri Hristamyan, Desislava Makakova-Tilova

**Affiliations:** 1 Medical College, Medical University of Plovdiv, 4002 Plovdiv, Bulgaria; 2 Clinic of Endocrinology and Metabolic Diseases, St. George University Hospital, Medical University of Plovdiv, 4002 Plovdiv, Bulgaria; 3 Department of Prosthetic Dentistry, Faculty of Dental Medicine, Medical University of Plovdiv, 4002 Plovdiv, Bulgaria; 4 Department of Epidemiology and Disaster Medicine, Faculty of Public Health, Medical University of Plovdiv, 4002 Plovdiv, Bulgaria

**Keywords:** obesity, psychological factors, perceived stress, resilience, aggression, young adults

## Abstract

**Background:** Obesity and overweight are escalating global public health concerns. This study examined the relationship between Body Mass Index (BMI) and psychological factors in young adults. **Methods:** A total of 283 participants aged 19–30 years were categorized by BMI (normal weight, overweight, obesity) using self-reported data. Psychological assessments included the Connor–Davidson Resilience Scale (CD-RISC), Perceived Stress Scale (PSS), Emotion Regulation Questionnaire (ERQ), and Buss–Perry Aggression Questionnaire (BPAQ), all with acceptable reliability. Individuals with psychiatric, neurological, or chronic somatic conditions were excluded. Analyses involved MANOVA, ANOVA, ANCOVA, and hierarchical regression, controlling for gender, physical activity, smoking, caffeine, alcohol, medication, and sleep disturbances. **Results:** MANOVA showed a near-significant multivariate effect of BMI on psychological variables (Wilks’ Lambda = 0.913, *p* = 0.068). ANOVA revealed significant differences in perceived stress among BMI groups (F (2, 279) = 3.796, *p* = 0.024), with obese participants reporting lower stress. Hierarchical regression identified perceived stress as the strongest predictor of BMI, followed by resilience and physical aggression. General aggression was negatively associated with BMI, suggesting potential compensatory mechanisms. **Conclusions:** Perceived stress and resilience showed small but significant associations with BMI in young adults, suggesting a modest contribution of psychological factors to weight-related health.

## 1. Introduction

Obesity and overweight have become significant global public health challenges, with their prevalence increasing dramatically over recent decades [1]. Obesity is characterized by excessive accumulation of body fat, often assessed using Body Mass Index (BMI), and is linked to numerous adverse health outcomes [2]. The World Health Organization (WHO) reports that approximately one in eight people globally lives with obesity, with prevalence having more than doubled since the 1990s, particularly among adolescents [3]. Projections indicate that the global prevalence of overweight and obesity may increase by approximately 30.7% over the next 30 years [4].

Obesity is consistently associated with a wide range of physical health conditions and is recognized as a modifiable risk factor for several chronic diseases, including certain cancers [5]. However, its impact extends beyond physical health, significantly affecting mental well-being. Obesity and mental health disorders share a complex, bidirectional relationship, particularly in adolescents. Excess body weight can predispose individuals to mental health impairments such as depression, anxiety, and reduced quality of life, while psychological difficulties can contribute to the development and persistence of obesity via behavioral and physiological pathways [6,7].

BMI is the most widely used method for assessing obesity in both clinical and public health settings, and recent research has highlighted the complex relationships between body weight and psychological processes in young adults. Higher BMI has been linked to elevated perceived stress [8], difficulties in emotion regulation [9,10], and lower psychological resilience [11]. Some studies also suggest that increased BMI may be associated with higher aggression levels, particularly when self-regulatory capacity is limited [12]. These findings underscore the importance of a multidisciplinary approach to understanding overweight and obesity in youth, integrating both biological and psychosocial dimensions.

Psychological resilience—the capacity to adapt to stress and adversity—has been identified as a protective factor against increased BMI [11,13]. Research among university populations indicates that individuals with higher resilience are more likely to engage in healthier dietary and behavioral patterns, potentially reducing the risk of being overweight. Interestingly, some community-based studies have reported paradoxical associations, with individuals exhibiting greater resilience sometimes presenting higher BMI, highlighting the complex interplay between mental strength and body weight regulation [14].

Conversely, chronic perceived stress has been consistently linked to increased BMI, often mediated by maladaptive coping strategies such as emotional eating and poor emotion regulation [15,16]. In particular, emotion suppression—a dysfunctional regulation style—has emerged as a strong predictor of emotional eating and subsequent weight gain [9,16]. Mental health disorders can further exacerbate obesity-related physical health risks through lifestyle and physiological mechanisms, including oxidative stress and systemic inflammation [17]. Unhealthy dietary patterns commonly associated with obesity, such as irregular meal consumption and poor nutrition, have also been linked to cognitive impairments and elevated risks of mental health problems, highlighting the interconnected nature of diet, obesity, and psychological well-being [18].

Behavioral dimensions such as hostility and aggression have been explored as potential psychosocial mediators of body weight. Studies among university students report positive correlations between higher BMI and manifestations of anger and hostility, especially under conditions of low self-control [12,19]. Similar patterns have been observed in multinational cohorts, suggesting that these psychophysiological mechanisms may be cross-culturally relevant.

Despite growing interest, existing studies often examine these psychological constructs in isolation, limiting the understanding of their combined effects on BMI. Methodological variability further challenges the generalizability of findings. The present study aims to address these gaps by simultaneously investigating perceived stress, psychological resilience, emotion regulation, and aggression concerning BMI among young adults.

This study aims to examine the relationships between BMI and multiple psychological factors—including perceived stress, psychological resilience, emotion regulation, and aggression—simultaneously in young adults. Based on previous research, the following hypotheses were formulated to guide the investigation:

**H_0_ (null hypothesis):**  *There is no significant association between BMI and perceived stress, aggression, or psychological resilience in young adults.*

**H_1_ (alternative hypothesis):**  *Higher BMI is positively associated with perceived stress and aggression and negatively associated with psychological resilience in young adults.*

Within this conceptual model, higher BMI is assumed to be associated with higher levels of subjective stress and aggression and with lower psychological resilience, with emotion-regulation strategies potentially acting as mediators or moderators of these relationships.

## 2. Materials and Methods

### 2.1. Participants

Participant recruitment and data collection were conducted between March 2022 and December 2024, following a cross-sectional study design. All questionnaires were administered in Bulgarian in a controlled academic environment. This timeframe refers solely to recruitment, confirming the design is not longitudinal.

Eligibility criteria required participants to be within the specified age range, fluent in Bulgarian, and able to complete psychometric questionnaires electronically. Individuals with severe psychiatric disorders (e.g., psychotic disorders), neurological conditions, or chronic somatic diseases impairing daily functioning were excluded. Participants who completed less than 85% of the assessment battery were also excluded.

BMI was calculated from self-reported height and weight using the standard formula [weight (kg)/height^2^ (m^2^)] and classified according to World Health Organization guidelines: normal weight (BMI 18.5–24.9), overweight (BMI 25.0–29.9), and obesity (BMI ≥ 30). BMI classification served as the basis for comparing psychometric outcomes across groups, focusing on perceived stress, resilience, emotion regulation, and aggression. Group allocation was automated based on BMI calculations, without subjective research input. A priori sample size estimation ensured sufficient statistical power and comparability across groups, based on effect sizes reported in prior studies.

To test whether psychological and behavioral variables predict BMI variation, hierarchical stepwise regression analyses were conducted. BMI served as the dependent variable, with predictors including psychological indicators (resilience, perceived stress, cognitive reappraisal, expressive suppression, anger, and general aggression) and demographic/behavioral covariates (sex, age, physical activity, smoking status, alcohol and caffeine consumption, and sleep disturbances). This approach allowed stepwise evaluation of each predictor’s independent contribution to BMI, controlling for other variables.

### 2.2. Study Procedures and Measures

Participants completed the questionnaires under standardized conditions within a fixed duration of one hour. They were instructed to respond spontaneously and without hesitation to minimize social desirability bias. A social desirability scale embedded in the survey identified 24 participants whose responses suggested potential bias; these participants were excluded.

Participants were fully informed about the study’s purpose, content, and duration, provided written consent, and were free to ask questions or withdraw at any time without consequence. The procedure posed no physical or psychological risk. No financial or material compensation was provided, and all data was collected solely for scientific purposes in compliance with national legislation and the General Data Protection Regulation (GDPR).

#### Anthropometric Measures

At the beginning of the study, participants provided self-reported data on height and body weight, which were used solely for preliminary screening purposes. For the statistical analyses, all anthropometric indicators were based exclusively on objective measurements. Body weight and body composition were assessed by an endocrinologist using a professional medical-diagnostic analyzer, Tanita (Tanita model TBF300A, Arlington Heights, IL, USA), operating on the principle of bioelectrical impedance analysis (BIA). Height was measured with a standard stadiometer, with participants standing upright and barefoot. Body Mass Index (BMI, kg/m^2^) was calculated from the objectively measured height and weight, and BMI categories (normal weight, overweight, obesity) were defined according to World Health Organization (WHO) criteria. Only objectively measured height and weight values—not the self-reported data—were used in all subsequent analyses to enhance the diagnostic accuracy of BMI and the overall reliability of the findings.

### 2.3. Psychometric Instruments

Resilience was assessed using the 10-item Connor–Davidson Resilience Scale (CD-RISC), rated on a 5-point Likert scale (0–4), with higher scores reflecting greater resilience. The scale conceptualizes resilience as the capacity to cope with stress, anxiety, and depressive symptoms. Internal consistency in the current sample was excellent (Cronbach’s α = 0.88). Perceived stress was measured using the Perceived Stress Scale (PSS), which evaluates the extent to which individuals perceive their lives as unpredictable, uncontrollable, and overwhelming over the past month. The scale showed acceptable reliability (α = 0.75). Emotion regulation was measured with the Emotion Regulation Questionnaire (ERQ), comprising two subscales: cognitive reappraisal and expressive suppression. Items assess individuals’ tendencies and perceived ability to manage emotional experiences and expressions. Internal consistency was good (α = 0.77).

Aggression was assessed using the culturally adapted and validated Bulgarian version of the Buss–Perry Aggression Questionnaire (AQ), comprising 29 items across four subscales: anger, physical aggression, verbal aggression, and hostility, as well as a total general aggression score. Reliability in this study was strong (α = 0.84).

### 2.4. Statistical Analysis

Descriptive statistics, analysis of variance (ANOVA), and multiple regression analyses were conducted using IBM SPSS Statistics for Windows, Version 23. ANOVA was applied to compare means across BMI categories, with statistical significance defined at α = 0.05.

Multiple regression analyses evaluated the predictive relationships between psychological variables (resilience, perceived stress, reappraisal, suppression, anger, and general aggression) and BMI. Models controlled for potential confounders, including age, sex, physical activity, smoking, alcohol and caffeine consumption, and sleep disturbances, ensuring independence and robustness of the estimated effects. Hierarchical stepwise regression allowed for the evaluation of each predictor’s unique contribution while accounting for covariates, aiming to identify a psychological profile associated with body mass variation in young adults.

## 3. Results

### 3.1. Characteristics of the Participants

The study sample consisted of 283 young adults aged 19–30 years (M = 22.25, SD = 5.54), with an almost equal gender distribution (140 males, 49.5%; 143 females, 50.5%). Participants were classified into three BMI categories: normal weight (*n* = 154, 54.4%), overweight (*n* = 80, 28.3%), and obese (*n* = 49, 17.3%). Mean age was similar across groups, ranging from 22.02 to 22.61 years. Body weight increased as expected across BMI classifications, with averages of 75.16 kg among normal-weight participants, 94.93 kg among overweight participants, and 101.55 kg among obese participants. Obese individuals reported the lowest levels of regular leisure-time physical activity, although total movement hours (including occupational activity) were slightly higher in the overweight and obese groups.

Most participants were urban residents of Plovdiv and surrounding areas and represented diverse educational and occupational backgrounds, including university students, employed adults, and unemployed individuals. Data were collected in a controlled university setting under researcher supervision between March 2022 and December 2024. All questionnaires were administered in Bulgarian, the participants’ native language.

Lifestyle behaviors showed modest variation across BMI groups. Smoking prevalence was highest among obese participants (40.3%), followed by those who were overweight (33.3%) and normal weight (32.3%). Mean daily cigarette consumption followed a similar pattern. Coffee and alcohol consumption showed slight group differences without a consistent trend. Sleep disturbances were reported by 21–33% of participants, with mean scores slightly lower among the obese group.

Overall, the sample was well-balanced across BMI, sex, and age, with modest variation in lifestyle behaviors, providing a suitable foundation for examining associations between BMI and psychological factors. Demographic and behavioral characteristics for each group are summarized in Table 1.

### 3.2. Multivariate Analysis of Variance (MANOVA)

To examine the influence of BMI on multiple psychological variables, a multivariate analysis of variance (MANOVA) was conducted. The three BMI groups—normal weight, overweight, and obese—were compared across several dependent variables: perceived stress (PSS), psychological resilience (CD-RISC), emotion regulation strategies (ERQ: reappraisal and suppression), and aggression dimensions (Buss–Perry Aggression Questionnaire: anger, physical aggression, verbal aggression, hostility, and general aggression). Covariates included gender, weekly physical activity, alcohol and caffeine consumption, smoking status, medication use, and sleep disturbances. Assumptions of multivariate normality and homogeneity of covariance matrices were verified before analysis. Multivariate tests indicated a near-significant effect of BMI on the combined psychological variables (Wilks’ Lambda = 0.913, F(16,544) = 1.584, *p* = 0.068; Roy’s Largest Root = 0.089, F(8273) = 3.045, *p* = 0.003), supporting subsequent univariate analyses.

Univariate ANOVAs revealed that perceived stress (PSS) was the only psychological variable significantly differing between BMI groups. Obese participants reported lower perceived stress (M = 47.76, SE = 0.336) compared to normal-weight (M = 48.16, SE = 0.313) and overweight participants (M = 48.36, SE = 0.346), with mean differences of −0.405 (*p* = 0.045, 95% CI [−0.804, −0.007]) and −0.604 (*p* = 0.042, 95% CI [−1.192, −0.015]), respectively. These differences remained significant after Bonferroni correction, suggesting a potential inverse association between BMI and perceived stress, possibly due to adaptive or defensive cognitive mechanisms among individuals with long-term obesity.

All other psychological variables—including resilience, cognitive reappraisal, suppression, and aggression dimensions—did not differ significantly across BMI groups (all *p* > 0.05). For example, CD-RISC scores were similar across groups (M ≈ 75.1–75.5, *p* = 1.000), ERQ reappraisal was slightly lower among obese participants (M = 29.32, *p* > 0.07), and aggression subscales showed no significant variation (*p* > 0.31).

A multivariate analysis of covariance (MANCOVA) was conducted to control for covariates. The multivariate effect of BMI remained significant (Wilks’ Lambda = 0.870, F(24, 789.484) = 1.611, *p* = 0.032, η^2^ = 0.045), indicating that psychological variation associated with BMI was not fully explained by the covariates. Additional tests (Pillai’s Trace = 0.133, Hotelling’s Trace = 0.144, Roy’s Largest Root = 0.102) supported the statistical significance of the effect, although the effect size was small (η^2^ = 0.045), which is expected given the complex and multifactorial nature of BMI. 

### 3.3. Multiple Linear Regression

A hierarchical stepwise multiple regression analysis was conducted to examine the predictive value of psychological variables on BMI as a continuous outcome. Four successive models were tested, introducing predictors incrementally to assess additional explanatory power (Table 2).

Model 1: Included perceived stress (PSS) only, which emerged as a significant negative predictor of BMI (β = −0.188, *p* = 0.001), accounting for 3.5% of BMI variance (R^2^ = 0.035, F(1, 281) = 10.319, *p* = 0.001).

Model 2: Added psychological resilience (CD-RISC), which positively predicted BMI (β = 0.168, *p* = 0.004). The explained variance increased to 6.4% (ΔR^2^ = 0.028, F Change = 8.396, *p* = 0.004).

Model 3: Introduced physical aggression (AQ_FA), which also positively predicted BMI (β = 0.169, *p* = 0.003), raising the total variance explained to 9.2% (R^2^ = 0.092, Adjusted R^2^ = 0.082; F Change = 8.784, *p* = 0.003).

Model 4: Added general aggression (AQ_GA), which showed a negative association with BMI (β = −0.207, *p* = 0.039). Total variance explained reached 10.6% (R^2^ = 0.106), with the model remaining statistically significant (F(4, 278) = 8.235, *p* < 0.001).

This proportion of explained variance is small and suggests that the psychological variables examined contribute only modestly—although statistically significantly—to BMI variation in young adults.

Across all models, perceived stress remained the most robust predictor (β range: −0.135 to −0.195, *p* < 0.035). Resilience and physical aggression maintained consistent positive associations with BMI, whereas general aggression exhibited an unexpected negative association, potentially reflecting compensatory behavioral patterns or intrapsychic conflict among individuals with higher BMI. Collinearity diagnostics indicated acceptable tolerance levels (T > 0.228), and residual analyses confirmed normality, homoscedasticity, and absence of influential outliers (Mahalanobis Distance and Cook’s Distance).

## 4. Discussion

The present study examined the associations between BMI and key psychological characteristics—subjective stress, resilience, emotion regulation, and aggression—in a sample of young adults. Rather than revealing a uniform pattern of associations, the findings indicate a heterogeneous profile in which only subjective stress and selected facets of aggression show statistically significant relationships with BMI. This pattern illustrates the limited psychological contribution to variations in body weight, which is expected given the multifactorial nature of obesity.

The lower levels of self-reported stress among participants with higher BMI constitute the most atypical finding and diverge from a substantial portion of the literature, where higher BMI is typically associated with elevated chronic stress. This discrepancy suggests the presence of contextual, age-specific, or behavioral characteristics unique to the studied population that may not have been fully captured by the employed measures. Similar inconsistencies were observed for resilience and general aggression, further suggesting that psychological mechanisms influencing body weight may manifest differently in early adulthood.

Overall, the null hypothesis (H_0_)—that there would be no significant association between BMI and most psychological variables—was accepted for all outcomes except perceived stress and physical aggression, which demonstrated significant associations. Notably, the direction of the association between perceived stress and BMI was opposite to the original prediction, indicating a more complex relationship than anticipated. These findings provide novel insights, particularly in the context of the global increase in overweight and obesity prevalence [20,21].

Regarding behavioral characteristics, participants with obesity exhibited the lowest average weekly physical activity, while no significant differences were observed between normal-weight and overweight groups. This is consistent with prior evidence indicating that insufficient leisure-time physical activity is associated with higher BMI categories [22]. Smoking prevalence was highest among participants with obesity, though existing studies remain mixed: some report that higher adolescent BMI increases the likelihood of adult smoking, especially among females [23], whereas others suggest an inverse relationship between smoking and BMI [24]. Sleep disturbances were least frequently reported among obese participants, contrasting with meta-analytic research showing that poor sleep quality and short sleep duration elevate the odds of overweight and obesity [25,26]. Coffee consumption was highest among participants with obesity, consistent with findings that the positive association between caffeine intake and muscle mass diminishes among individuals with elevated BMI [27]. Alcohol use was most frequent among normal-weight participants, although larger epidemiological surveys indicate that higher alcohol intake is associated with increased obesity risk [28].

The primary significant psychological finding was that perceived stress differed across BMI groups. Obese participants reported lower perceived stress compared to normal-weight and overweight individuals, which contrasts with prior research highlighting a positive association between BMI and chronic stress [29,30]. This result may reflect psychological adaptation mechanisms—such as denial or cognitive distancing—that reduce subjective stress perception [31,32]. Multiple regression analysis further indicated that lower perceived stress significantly predicted higher BMI, suggesting a potential compensatory or reverse relationship, possibly mediated by emotion-driven eating or metabolic adaptation [33,34]. Accordingly, the null hypothesis for perceived stress was rejected, although the association was in the opposite direction to the original hypothesis.

Psychological resilience (CD-RISC) and general aggression (AQ_GA) did not show the expected relationships with BMI. Resilience positively predicted BMI, contrary to the hypothesized negative association, while general aggression negatively predicted BMI, opposite to the predicted positive association. Emotion regulation strategies and other aggression dimensions were not significantly associated with BMI; therefore, the null hypothesis (H_0_) was accepted for these constructs. Physical aggression (AQ_FA) showed a positive association with BMI, partially supporting the original hypothesis and resulting in a partial rejection of H_0_.

The observed positive predictive contributions of psychological resilience and physical aggression to elevated BMI challenge the conventional assumption that greater resilience is uniformly associated with better physical health outcomes [35]. One possible interpretation is that individuals with higher BMI may develop coping strategies that help maintain psychological well-being despite increased somatic risks. However, this interpretation remains speculative, and causal mechanisms cannot be inferred from cross-sectional data.

The negative association between general aggression and BMI was also unexpected. It may reflect internalized or inhibited expressions of anger, potentially manifesting in maladaptive behaviors such as emotional eating; however, this explanation is highly tentative. The study did not assess underlying psychological processes—such as emotion-driven eating, anger suppression, or intrapsychic conflict—and therefore these hypotheses should be viewed as theoretical possibilities rather than validated mechanisms.

Other psychological indicators, including emotion regulation strategies and additional aggression dimensions, did not differ significantly across BMI categories, suggesting relative psychological homogeneity in these domains. This pattern aligns with prior research indicating that not all aspects of psychological functioning are equally influenced by body weight [36].

In the context of rising global obesity prevalence, particularly in low- and middle-income countries [37,38], and the substantial comorbidity between obesity and mental health disorders [39], these findings highlight the need for integrated interventions that address both physical and psychological health. The results also underscore the importance of considering directionality and compensatory mechanisms when interpreting associations between BMI and psychological variables.

Although statistically significant, the observed associations were small in magnitude, and the regression model’s overall explained variance was modest (R^2^ = 0.106). This suggests that the psychological variables examined account for only a limited proportion of BMI variability in young adults, with behavioral, biological, and environmental determinants likely playing a more substantial role.

From a clinical and preventive perspective, the findings suggest that weight-management interventions for young adults may benefit from concurrently assessing and addressing subjective stress and psychological resilience, even if their statistical contributions to BMI variation are modest. In a university context, this could involve incorporating brief screening tools and psychoeducational modules focused on stress management and adaptive coping into broader healthy-lifestyle programs, potentially supporting both weight-related outcomes and the overall psychological well-being of students.

### 4.1. Limitations

Several methodological limitations should be acknowledged. First, the cross-sectional design precludes any causal inference regarding the directionality of the relationships observed. Second, BMI was calculated using self-reported height and weight, which may be prone to systematic underestimation of body weight and overestimation of height, potentially reducing the accuracy of BMI classification. Third, the sample consisted exclusively of young adults from a single university in Plovdiv, Bulgaria, limiting the generalizability of the findings to other age groups and sociocultural contexts. Fourth, several important confounding variables—such as dietary habits, socioeconomic status, family history of obesity, hormonal or contraceptive factors, and mental health indicators (e.g., depression, anxiety, body image, weight stigma)—were not assessed. The absence of these variables increases the likelihood of residual confounding and may partly account for the modest effect sizes observed. Finally, the sample consisted exclusively of young adults aged 19–30 years, which limits generalizability to other age cohorts. Psychological responses to body weight may differ among adolescents, middle-aged, and older adults due to developmental, hormonal, and sociocultural factors influencing both self-perception and coping strategies. Additionally, the sample consisted exclusively of young adult students from a single university in Plovdiv, representing a relatively homogeneous age and sociocultural context. This limits the generalizability of the findings to other age groups and to non-student populations. Moreover, several important socioeconomic and medical variables—such as income, dietary habits, family history of obesity, use of hormonal contraception, and other hormonal factors—were not included in the models. The absence of these covariates likely contributes to the low explanatory power of the regression model (R^2^ = 0.106), as a substantial proportion of BMI variation is expected to relate to unmeasured biological and behavioral determinants.

### 4.2. Future Directions

Future studies should integrate interoceptive and physiological indicators—such as heart rate variability (HRV), vagal tone, and interoceptive accuracy—as potential mediators linking psychological variables with BMI. Prioritizing longitudinal and mixed-method designs will be essential for identifying causal pathways and clarifying whether these associations operate bidirectionally or in cyclical patterns. Broadening the scope of inquiry to include additional psychological constructs—such as self-concept, stigma internalization, and emotional eating—may further elucidate mechanisms through which emotional and behavioral regulation contribute to body weight variation [13].

Cross-cultural and lifespan research is also warranted to determine whether the observed patterns generalize across diverse populations and socio-demographic contexts. Integrating physiological markers (e.g., cortisol, inflammatory cytokines, metabolic indicators) with psychosocial assessments could provide deeper insight into the biopsychosocial processes underlying weight regulation. Such multidisciplinary approaches would meaningfully advance theoretical models of obesity and support the development of holistic interventions that promote both physical and psychological well-being [16,40].

## 5. Conclusions

The present study underscores the complex interplay between Body Mass Index (BMI) and psychological factors in young adults. The inverse association observed between perceived stress and BMI suggests that individuals with obesity may report lower levels of subjective stress, potentially reflecting specific forms of psychological adaptation to chronically elevated body weight. However, this interpretation remains speculative and requires confirmation through future research incorporating more precise psychological and physiological assessments. The present findings should therefore be viewed as exploratory and hypothesis-generating. Given the small effect sizes and limited explained variance, the practical implications for clinical or public health interventions are modest. Psychological constructs such as perceived stress and resilience may hold value as secondary indicators for screening or monitoring within comprehensive weight-management programs, but they should not be considered primary determinants of BMI. Continued longitudinal research is needed to validate these associations and to clarify their stability over time. Psychological resilience and physical aggression emerged as positive predictors of BMI, highlighting the potential roles of both adaptive and maladaptive psychological traits in weight regulation. These results indicate that body weight is shaped not only by behavioral and physiological mechanisms but also by emotional and cognitive coping processes. Although the proportion of variance explained was moderate, the predictive model demonstrated statistical robustness and theoretical relevance within an exploratory framework. Overall, the findings reinforce the importance of adopting a biopsychosocial approach to understanding obesity. Integrating psychological, behavioral, and lifestyle factors may provide a more comprehensive perspective on the mechanisms linking psychological functioning and body weight during young adulthood.

## Figures and Tables

**Table 1:** Distribution of characteristics of the study groups by BMI categories.

Characteristic	Normal Weight (n = 154, 54.4%)	Overweight (n = 80, 28.3%)	Obese (n = 49, 17.3%)
Sex—Males (%)	23.0%	14.8%	11.7%
Sex—Females (%)	31.4%	13.4%	5.7%
Mean Age (years)	22.02 (SD = 5.59, SE = 0.45)	22.61 (SD = 5.81, SE = 0.83)	22.37 (SD = 5.59, SE = 0.45)
Mean Body Weight (kg)	75.16 (SD = 11.49, SE = 0.93)	94.93 (SD = 20.17, SE = 3.11)	101.55 (SD = 23.01, SE = 3.55)
Mean Weekly Physical Activity (hours)	12.59 (SD = 11.68, SE = 0.94)	14.33 (SD = 14.80, SE = 1.66)	14.86 (SD = 14.94, SE = 2.14)
Smokers (%)	32.3%	33.3%	40.3%
Mean Cigarettes per Day	4.91 (SD = 9.17, SE = 0.74)	4.95 (SD = 7.89, SE = 0.88)	6.24 (SD = 8.86, SE = 1.27)
Coffee Consumers (%)	36.3%	21.6%	42.1%
Mean Coffee Consumption (cups/day)	1.53 (SD = 1.31, SE = 0.11)	1.30 (SD = 1.23, SE = 0.14)	1.78 (SD = 1.31, SE = 0.19)
Alcohol Consumers (%)	57.5%	35.6%	41.3%
Mean Alcohol Consumption (units/week)	2.91 (SD = 1.34, SE = 0.11)	2.16 (SD = 1.53, SE = 0.17)	2.43 (SD = 1.37, SE = 0.20)
Sleep Disturbances (%)	33.3%	31.4%	21.4%
Mean Sleep Disturbance Score	1.82 (SD = 0.40, SE = 0.03)	1.79 (SD = 0.41, SE = 0.05)	1.69 (SD = 0.59, SE = 0.08)

SD = standard deviation; SE = standard error; BMI = body mass index.

**Table 2:** Multiple linear regression.

Model	Predictor	B	SE B	β (Beta)	t	*p*	ΔR^2^	R^2^	Adj. R^2^	F Change	Sig. F Change
1	(Constant)	30.203	2.148	—	14.062	<0.001	0.035	0.035	0.032	10.319	0.001
	PSS	−0.143	0.044	−0.188	−3.212	0.001					
2	(Constant)	26.058	2.558	—	10.188	<0.001	0.028	0.064	0.057	8.396	0.004
	PSS	−0.142	0.044	−0.188	−3.251	0.001					
	CD-RISK	0.055	0.019	0.168	2.898	0.004					
3	(Constant)	23.972	2.619	—	9.153	<0.001	0.029	0.092	0.082	8.784	0.003
	PSS	−0.148	0.043	−0.195	−3.423	0.001					
	CD-RISK	0.058	0.019	0.177	3.093	0.002					
	AQ_FA	0.096	0.032	0.169	2.964	0.003					
4	(Constant)	24.686	2.626	—	9.399	<0.001	0.014	0.106	0.093	4.305	0.039
	PSS	−0.102	0.048	−0.135	−2.121	0.035					
	CD-RISK	0.048	0.019	0.147	2.514	0.013					
	AQ_FA	0.183	0.053	0.322	3.465	0.001					
	AQ_GA	−0.051	0.025	−0.207	−2.075	0.039					

B = Unstandardized regression coefficient; SE B = Standard error of B; β = Standardized regression coefficient; t = t-statistic; p = Significance value (*p*-value); ΔR^2^ = Change in coefficient of determination; R^2^ = Coefficient of determination; Adj. R^2^ = Adjusted coefficient of determination; F Change = F statistic for model change; Sig. F Change = Significance of F change.

## Data Availability

The data presented in this study are available on request from the corresponding author due to ethical and privacy restrictions related to participant confidentiality.

## Abbreviations

The following abbreviations are used in this manuscript:

BMI	Body Mass Index
WHO	World Health Organization
CD-RISC	Connor–Davidson Resilience Scale
PSS	Perceived Stress Scale
ERQ	Emotion Regulation Questionnaire
AQ	Buss–Perry Aggression Questionnaire
ANOVA	Analysis of Variance
MANOVA	Multivariate Analysis of Variance
ANCOVA	Analysis of Covariance
SE	Standard Error
SD	Standard Deviation
OR	Odds Ratio
CI	Confidence Interval
R^2^	Coefficient of Determination
β	Beta coefficient (standardized regression coefficient)
F Change	Change in F-statistic (in regression modeling)
T	Tolerance (collinearity diagnostics)
GDPR	General Data Protection Regulation
